# Mechanistic insights of O-GlcNAcylation that promote progression of cholangiocarcinoma cells via nuclear translocation of NF-κB

**DOI:** 10.1038/srep27853

**Published:** 2016-06-13

**Authors:** Chatchai Phoomak, Kulthida Vaeteewoottacharn, Kanlayanee Sawanyawisuth, Wunchana Seubwai, Chaisiri Wongkham, Atit Silsirivanit, Sopit Wongkham

**Affiliations:** 1 Department of Biochemistry, Faculty of Medicine, Khon Kaen University, Khon Kaen, 40002, Thailand; 2 Liver Fluke and Cholangiocarcinoma Research Center, Faculty of Medicine, Khon Kaen University, Khon Kaen, 40002, Thailand; 3 Department of Forensic Medicine, Faculty of Medicine, Khon Kaen University, Khon Kaen, 40002, Thailand

## Abstract

O-GlcNAcylation, an O-linked protein glycosylation with a single molecule of N-acetylglucosamine (GlcNAc), is reversibly controlled by O-GlcNAc transferase (OGT) and N-acetyl D-glucosaminidase (OGA). Aberrant O-GlcNAcylation contributes an important role in initiation and progression of many human cancers. Elevation of O-GlcNAcylation in tumor tissues and poor prognosis of cholangiocarcinoma (CCA) patients have been reported. In this study, the role of O-GlcNAcylation in promoting tumor progression was further investigated in CCA cell lines. Suppression of O-GlcNAcylation using small interfering RNAs of OGT (siOGT) significantly reduced cell migration and invasion of CCA cells whereas siOGA treated cells exhibited opposite effects. Manipulating levels of O-GlcNAcylation did affect the nuclear translocation of NF-κB and Akt-phosphorylation together with expression of matrix-metalloproteinases (MMPs). O-GlcNAcylation and nuclear translocation of NF-κB, the upstream signaling cascade of MMP activation were shown to be important for MMP activation. Immunoprecipitation revealed the elevation of O-GlcNAc-modified NF-κB with increased cellular O-GlcNAcylation. Involvement of O-GlcNAcylation in MMP-mediated migration and invasion of CCA cells was shown to be via O-GlcNAcylation and nuclear translocation of NF-κB. This information indicates the significance of O-GlcNAcylation in controlling the metastatic ability of CCA cells, hence, O-GlcNAcylation and its products may be new targets for treatment of metastatic CCA.

O-GlcNAcylation, an O-linked glycosylation of many nucleocytoplasmic proteins with a single molecule of *N*-acetylglucosamine (GlcNAc), is a dynamic and reversible process for adding or removal of GlcNAc onto a serine (Ser)/threonine (Thr) residue^1^. This process is regulated by the actions of two enzymes; O-GlcNAc transferase (OGT) and N-acetyl D-glucosaminidase (OGA), which catalyze O-GlcNAc addition and removal^2^,^3^.

GlcNAc or phosphate competitively modifies the same or proximal Ser/Thr residues of several nucleocytoplasmic proteins, leading to the changes in activity or conformation of the proteins^4^,^5^,^6^. As a consequence, O-GlcNAcylation of a particular protein plays a significant role on its destination and function, such as phosphorylation, degradation, localization, and protein-protein interaction^7^. Therefore, O-GlcNAcylation is involved in a wide range of biological processes, such as signal transduction, transcription, cell cycle regulation, and metabolism^6^,^7^,^8^,^9^. Alteration of O-GlcNAcylation has been shown to participate in the pathological progression of many human diseases^4^,^6^,^7^,^10^ including cancers. Elevations of O-GlcNAcylation have been demonstrated to be associated with the aggressiveness of many types of cancer; *e*.*g*., cancers of breast^11^,^12^, colon^13^,^14^, liver^15^, lung^13^, prostate^16^,^17^, pancreas^18^, and ovary^19^.

Cholangiocarcinoma (CCA) is a rare bile duct cancer, however, the incidence of CCA is now increasing worldwide. CCA is highly metastatic which is the main cause of death of CCA patients. Understanding the molecular basis of metastasis may lead to the discovery of alternative or adjunctive and effective treatment for CCA. The elevation of O-GlcNAcylation in CCA tissues of patients was recently reported and shown to be the result of increasing OGT and decreasing OGA^20^. Moreover, high levels of O-GlcNAcylation in CCA tissues were associated with poor clinical outcomes of the patients.

In this study, the association of O-GlcNAcylation and metastasis in CCA cell lines was emphasized. Suppression of O-GlcNAcylation significantly reduced cell migration and invasion of CCA cells whereas enhancing O-GlcNAcylation exhibited the opposite effects. A molecular mechanism by which O-GlcNAcylation regulated progression of CCA cells was revealed to be via O-GlcNAcylation of NF-κB. This information indicates the significant roles of O-GlcNAcylation in controlling the metastatic ability of CCA cells and hence O-GlcNAcylation and its products may be the new targets for treatment of CCA with metastasis.

## Results

### O-GlcNAcylation regulates migration and invasion abilities of CCA cells

**Figure 1:**
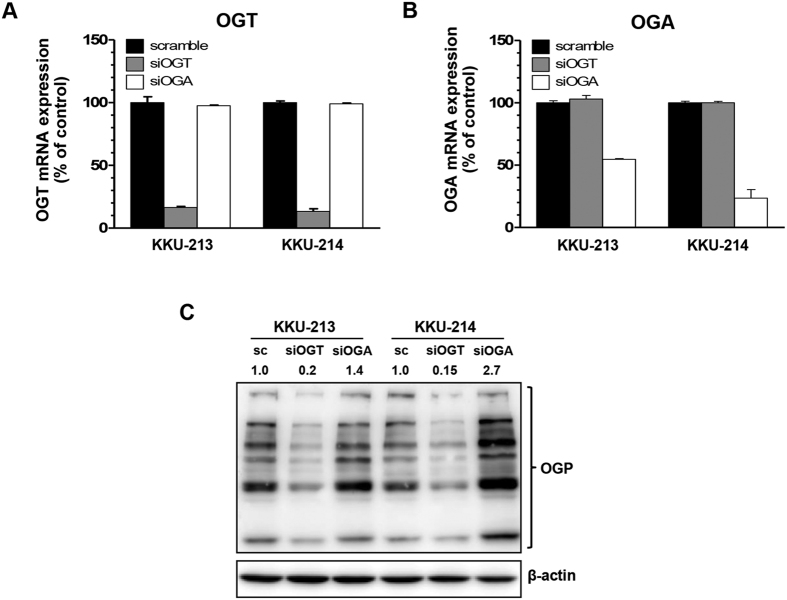
siOGT and siOGA could regulate O-GlcNAcylation in CCA cells. To manipulate the O-GlcNAcylation status in CCA cell lines, KKU-213 and KKU-214 cells were treated with siOGT or siOGA for 48 h. The treated cells were monitored for (**A**) OGT and (**B**) OGA expressions using real-time PCR; (**C**) O-GlcNAcylated products (OGP) using western blotting with O-GlcNAc-specific mAb (RL2). The numbers on top of the western blot represent the percentage of OGP by giving the scramble controls (sc) = 1. (**A**,**B**) are the averages from three independent experiments; (**C**) is one representative from three independent experiments.

As O-GlcNAcylation, is a dynamic and reversible process regulated by OGT (addition of O- GlcNAc) and OGA (removal of O-GlcNAc), therefore, cellular O-GlcNAcylation levels can be manipulated using small interfering RNAs specific to OGT or OGA. Suppression of OGT will decrease the O-GlcNAcylation whereas suppression of OGA will increase the O-GlcNAcylation and reverse the results of OGT suppression. In this study, the roles of O-GlcNAcylation in CCA were examined in two CCA cell lines, KKU-213 and KKU-214 using the specific siRNA to OGT (GCGUGUUCCCAAUAGUGUAtt) or OGA (GUCCACAGAUGGCUCUAAAtt)^15^. After 48 h of siRNA treatment, when compared to the scramble control cells, the OGT expression was decreased to 16% in KKU-213 and 13% in KKU-214 by siOGT (Fig. 1A) and OGA expression was suppressed by siOGA to 55% in KKU-213 and 24% in KKU-214 cells (Fig. 1B). Each siRNA had no cross-effect on the other, as cells treated with siOGT had no effect on OGA expression and *vice versa*. In addition, the cellular O-GlcNAcylation represented as OGP in the western blot, was significantly suppressed by siOGT and enhanced by siOGA (Fig. 1C). Compared with the scramble control, OGP was dramatically reduced to 0.2 in KKU-213 and 0.15 in KKU-214 by siOGT and was obviously increased to 1.4 in KKU-213 and 2.7 in KKU-214 by siOGA.

**Figure 2:**
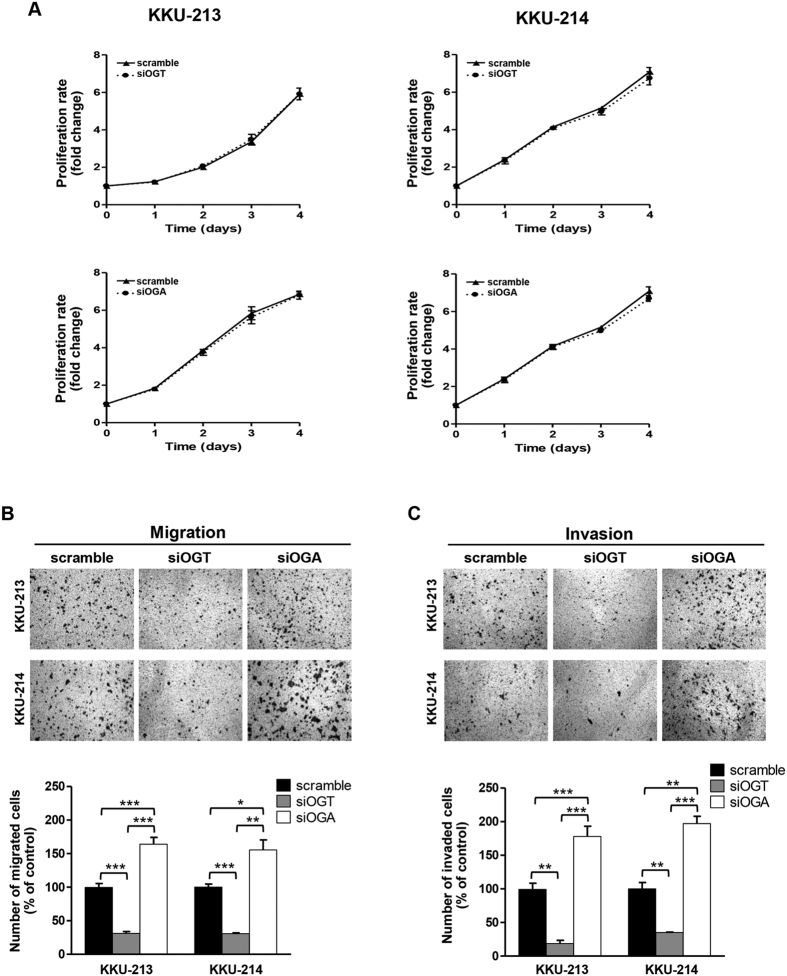
O-GlcNAcylation status modulated migration and invasion abilities of CCA cells. Treatment of siOGT or siOGA in CCA cell lines, KKU-213 and KKU-214, for 48 h had no effect on (**A**), cell proliferation, but significantly affected (**B**) cell migration and (**C**) invasion. The numbers of migrated and invaded cells were compared with the scramble controls (100%). The results (mean ± SEM) are the averages from three independent experiments; *P < 0.05, **P < 0.01, ***P < 0.001.

The effects of O-GlcNAcylation on cell proliferations of KKU-213 and KKU-214 cells were examined. As shown in Fig. 2A, neither treatments of siOGT or siOGA affected rates of cell proliferation. In contrast, siOGT treatment for 48 h, significantly reduced cell migration and invasion as determined by the Boyden chamber assay. Suppression of O-GlcNAcylation using siOGT could markedly decrease migratory cells to 30% (Fig. 2B), and invaded cells to 15–30% of the controls (Fig. 2C). Conversely, enhancement of O-GlcNAcylation by siOGA treatment significantly increased migration and invasion abilities to 150–180% of the controls (Fig. 2B,C).

### O-GlcNAcylation modulates expressions of matrix metalloproteases

**Figure 3:**
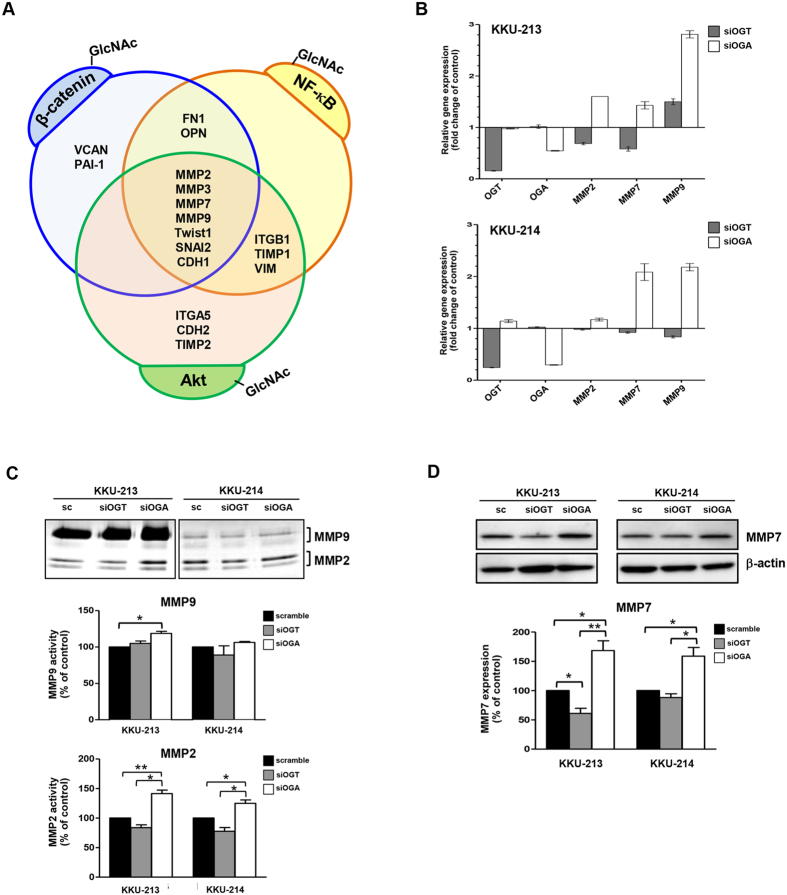
O-GlcNAcylation modulated progression of CCA cells via up-regulation of MMPs. The metastatic-associated genes were determined in 48 h with treated siOGT; and siOGA cells using real-time RT-PCR. (**A**) Seventeen metastatic-associated genes are grouped according to their up-stream O-GlcNAcylated regulators. (**B**) The expressions of *MMP2*, *MMP7* and *MMP9* were examined in siOGT and siOGA treated cells and compared with the scramble control cells using real-time PCR. (**C**) Validation of MMP2 and MMP9 by gelatin zymography and (**D**) of MMP7 by western blotting. The quantitative analyses are compared in each graph by using the scramble controls as 100%. The results (mean ± SEM) are the averages from three independent experiments; *P < 0.05, **P < 0.01.

To explore the molecular mechanism by which O-GlcNAcylation regulates migration and invasion of CCA cells, the expression of 17 metastasis associated genes reported to be the downstream signaling cascades of three O-GlcNAcylated proteins, namely Akt, β-catenin and NF-κB (Fig. 3A), that were determined in siOGT and siOGA treated cells. The expression levels of E-cadherin (CDH1), fibronectin (FN1), integrin-α5 (ITGA5), integrin-β1 (ITGB1), MMP2, MMP3, MMP7, MMP9, N-cadherin (CDH2), osteopontin (OPN), serpine 1 (PAI-1), slug (SNAI2), TIMP1, TIMP2, TWIST1, versican (VCAN), and vimentin (VIM) were examined using real time PCR, in KKU-213 cells treated with siRNA for 48 h. Genes with expression levels over 1.2 or less than 0.8 of the scramble control cells were considered to be significantly different. Genes in which expression levels were altered by siOGT treatment and reversed by siOGA treatment were selected for validation in KKU-214 cells (Supplementary Table S2). Finally, the expression levels of matrix metalloproteases (MMPs), namely MMP2, MMP7, and MMP9, were found to be suppressed. The expression levels of MMPs corresponded well with the levels of O-GlcNAcylation manipulated in both cell lines tested which indicated the strong association of MMPs and O-GlcNAcylation, therefore, the expressions of these MMPs genes were selected for further validation at the protein level. The association of O-GlcNAcylation and expression of MMPs was confirmed using the gelatin zymography assay. The activities of MMP2 were significantly reduced in siOGT and enhanced in siOGA treated cells, in both cell lines (Fig. 3C). Similar observations were obtained for MMP7 as shown by western blotting of both cell lines (Fig. 3D). To affirm the effect of O-GlcNAcylation on promoting progression of CCA cells, the consequences of increasing O-GlcNAcylation using PUGNAc (a well-known OGA inhibitor) were studied in one more CCA cell line, KKU-100. Comparable results similar to using siOGA were obtained (Supplementary Fig. S1). Taken together, the expressions of MMP2 and MMP7 exhibited obvious and consistent associations with the levels of O-GlcNAcylation in all CCA cell lines.

### Nuclear translocation of NF-κB and phosphorylation of Akt are regulated by O-GlcNAcylation

**Figure 4:**
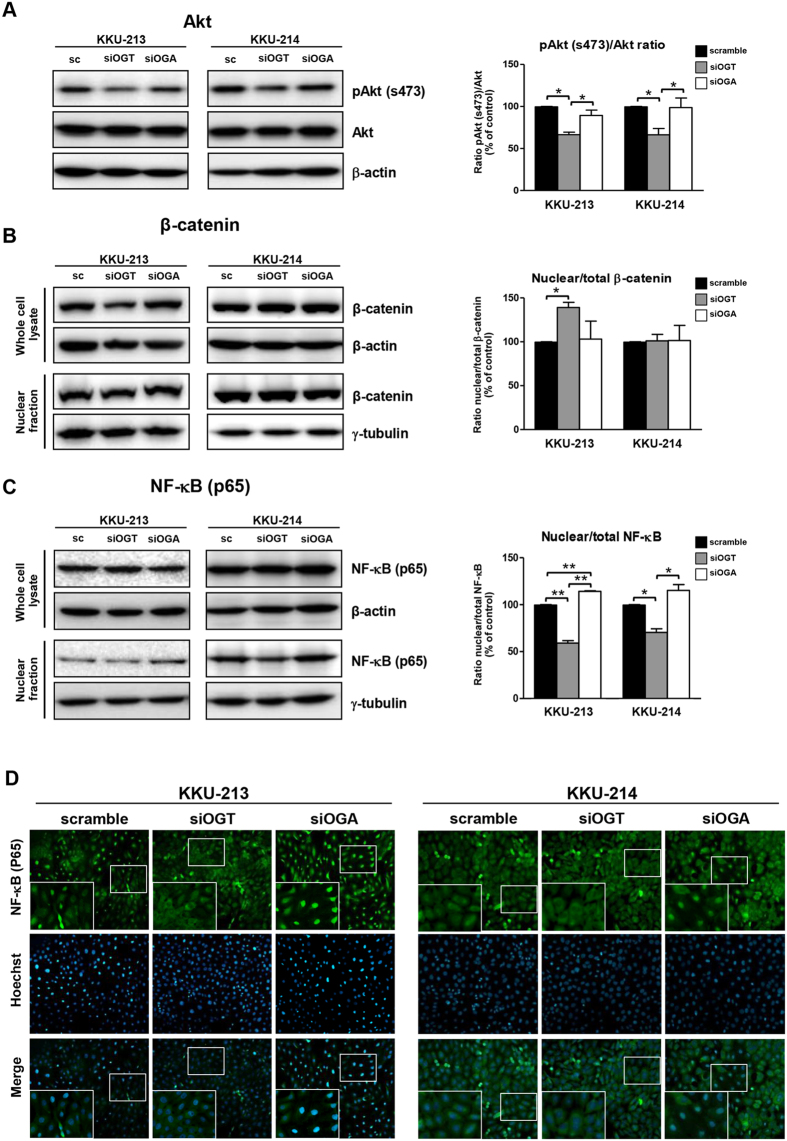
NF-κB nuclear translocations and Akt phosphorylations were associated with O-GlcNAcylation. The activations of Akt, β-catenin and NF-kB were determined in 48 h treated siOGT; and siOGA cells. (**A**) Total Akt and phosphorylated-Akt were determined by western blot analysis. (**B**) Total cellular and nuclear β-catenin and (**C**) NF-κB were examined by western blotting. (**D**) Cellular localization of NF-κB (green) is demonstrated by immunocytofluorescent staining; nuclei (blue) are stained with Hoechst 33342. The quantitative analyses are compared in each graph by using the scramble control as 100%. The results (mean ± SEM) are the averages from three independent experiments; *P < 0.05, **P < 0.01.

To understand the linkage of O-GlcNAcylation and MMPs expression, the upstream signaling cascades of MMP activation were further defined. As shown in Fig. 3A, PI3K-Akt, β-catenin, and NF-κB are the three suggested candidates. To demonstrate the association of these three candidates and the expression of MMPs, the activation of Akt phosphorylation (Ser473), and nuclear translocation of β-catenin and NF-κB (p65) were evaluated in the siOGT and siOGA-treated KKU-213 and KKU-214 cells. As shown in Fig. 4A, suppression of O-GlcNAcylation using siOGT effectively diminished phosphorylation of Akt in both CCA cell lines. The alteration of O-GlcNAcylation, however, did not affect the nuclear localization of β-catenin as β-catenin in the nuclear fraction was not altered by the manipulation of siOGT or siOGA (Fig. 4B). On the other hand, regulation of O-GlcNAcylation via siOGT or siOGA did affect the nuclear translocation of NF-κB. As compared to the scramble control cells, siRNA treatment did not disturb the level of total cellular NF-κB. In contrast, the nuclear NF-κB signal was significantly reduced in siOGT treated cells and opposite effects were observed in the siOGA treated cells (Fig. 4C).

To confirm the effect of O-GlcNAcylation on nuclear translocation of NF-κB, the immunocytofluorescent staining of NF-κB was examined in siOGT and siOGA-treated cells. As shown in Fig. 4D, most of the siOGT-treated cells exhibited cytoplasmic NF-κB staining, whereas most of the siOGA-treated cells possessed nuclear NF-κB signals.

### Nuclear translocation of NF-κB is associated with migration/invasion and MMP7 activity of CCA cells

**Figure 5:**
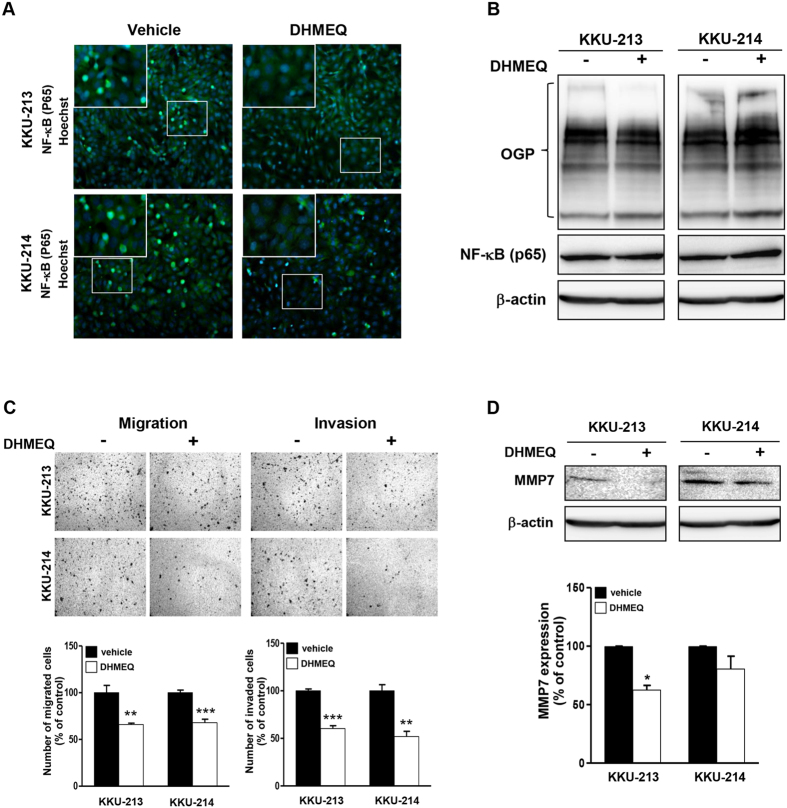
Migration and invasion of CCA cells were reduced by DHMEQ, a specific inhibitor of nuclear translocation of NF-κB. (**A**) The inhibition effect of DHMEQ on the nuclear translocation of NF-κB was confirmed by immunocytofluorescence. (**B**) DHMEQ had no effect on cellular OGP and NF-κB as determined by western blotting. (**C**) DHMEQ inhibited cell migration and invasion compared with the vehicle control cells. (**D**) Western blotting indicates that MMP7 expression was suppressed in the presence of DHMEQ. The data (mean ± SEM) in (**A**,**B**) represent one of two independent experiments. The quantitative analyses are compared in each graph by using the vehicle control as 100%. *P <  0.05, **P < 0.01, ***P < 0.001.

To test whether the nuclear translocation of NF-κB was related to migration and invasion activities of CCA cells, dehydroxymethylepoxyquinomicin (DHMEQ), a specific inhibitor of nuclear translocation of NF-κB^21^, was used for this purpose. The effectiveness of DHMEQ on the inhibition of nuclear translocation of NF-κB was checked first. The immunocytofluorescent staining of NF-κB demonstrated that DHMEQ did decrease the nuclear translocation of NF-κB in the DHMEQ-treated CCA cells (Fig. 5A). On the other hand, DHMEQ treatment did not affect the global O-GlcNAcylation or disturb the cellular expression of NF-κB as determined by western blots (Fig. 5B). The effects of DHMEQ on migration and invasion activities of CCA cells were assessed next. The numbers of migrated and invaded cells were significantly reduced in the DHMEQ treated cells compared with the vehicle control cells (Fig. 5C). The DHMEQ treatment also suppressed the expression of MMP7 as shown by western blot (Fig. 5D). Taken together, nuclear translocation of NF-κB had a positive correlation with migration/invasion and MMP7expression of CCA cell lines.

### NF-κB was modified by O-GlcNAc and modulated by cellular O-GlcNAcylation

**Figure 6:**
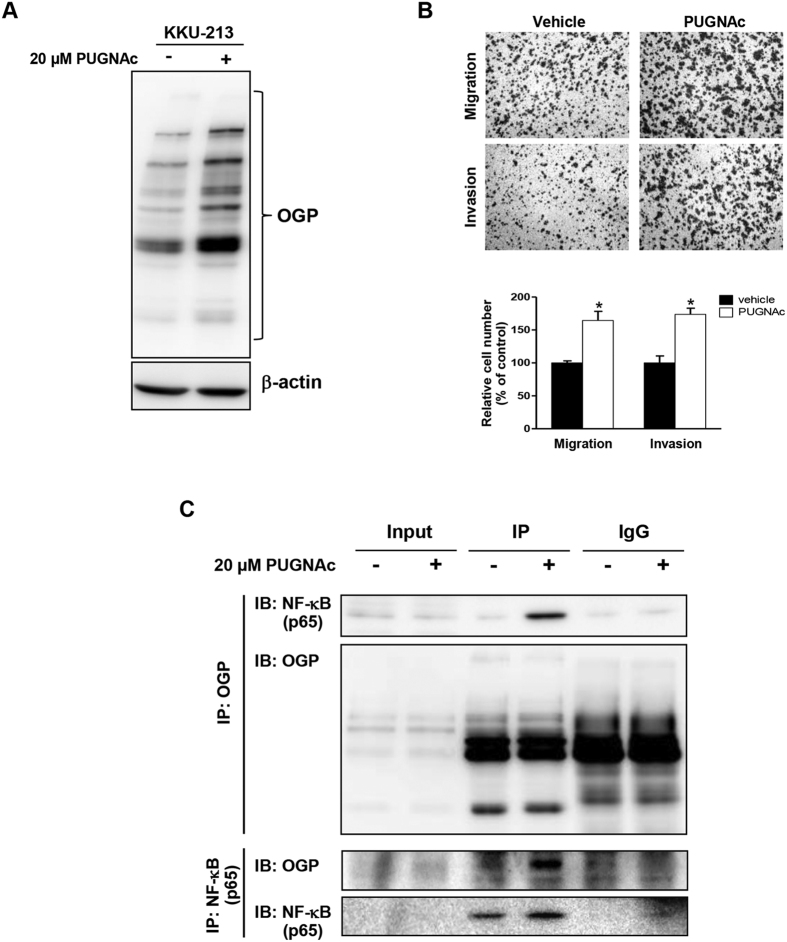
NF-κB was O-GlcNAcylated and modulated by cellular O-GlcNAcylation. (**A**) Western blot of OGP demonstrates the enhanced O-GlcNAcylation in KKU-213 cells using 20 μM PUGNAc. (**B**) The migration and invasion abilities of PUNAc treated cells were increased compared with vehicle control cells. The data shown are one representative from two independent experiments. (**C**) Cell lysates from cells treated (+) or untreated (−) with PUGNAc were subjected to immunoprecipitation using anti-OGP followed by western blot analysis with anti NF-κB; the *vice versa* experiments were also performed. The data are one representative from three independent experiments. The quantitative analyses (mean ± SEM) are compared in each graph by using the vehicle control as 100%. *P < 0.05, **P < 0.01, ***P < 0.001.

In this study, it was shown that O-GlcNAcylation did affect the nuclear translocation of NF-κB which in turn regulated cell migration and invasion via MMP expression. To affirm that NF-κB was O-GlcNAcylated, the immunoprecipitation of NF-κB (p65) was performed and verified with western blotting against O-GlcNAc specific mAb (RL2). The reciprocal immunoprecipitations of O-GlcNAcylated proteins with O-GlcNAc specific mAb and verified with NF-κB western blotting were also performed. To enhance the O-GlcNAcylation in CCA cells, cells were first treated with PUGNAc before the immunoprecipitation assay. The efficiency of PUGNAc on OGP enrichment was demonstrated by OGP western blot as shown in Fig. 6A. The migration and invasion of PUGNAc treated cells were observed as compared with vehicle control cells. PUGNAc treatment significantly induced the migration and invasion abilities of CCA cells similar to the siOGA treatment (Fig. 6B).

The specificity of the antibodies used in the immunoprecipitation assay were clarified using cell lysates treated with isotype-immunoglobulin as controls. As shown in Fig. 6C, NF-κB was identified in the immunoprecipitation of O-GlcNAcylated proteins from PUGNAc treated cells. In parallel, O-GlcNAc modification was identified in the immunoprecipitate of NF-κB. These results indicated that NF-κB was modified by O-GlcNAc and cellular O-GlcNAcylation modulated the level of O-GlcNAc-modified NF-κB.

## Discussion

Evidence from many studies indicates the positive association of O-GlcNAcylation and progression of cancer through *e*.*g*., growth, apoptosis resistance, migration, invasion, and angiogenesis of cancer^22^,^23^,^24^. Recently, elevation of O-GlcNAcylation in tumor tissues of CCA patients was reported in association with poor clinical outcomes^20^. In the current study, the association of O-GlcNAcylation with progression, migration and invasion, of CCA and the insights underlining the mechanisms are reported.

In this current study, specific siOGT and siOGA were used to suppress or enhance the O-GlcNAcylation status in two CCA cell lines. The involvement of O-GlcNAcylation and progression of CCA cells were demonstrated by the fact that suppression of O-GlcNAcylation using siOGT significantly decreased migration and invasion of CCA cell lines and the opposite observations were obtained when cells were treated with siOGA. This finding seems to be a shared feature of cancer cells. The association of O-GlcNAcylation and metastasis has been reported in many types of cancers. The *in vitro* functional analysis of HepG2, a hepatoma cell line, showed that migration and invasion abilities of the cells were significantly decreased after suppression of O-GlcNAcylation by siOGT and were increased after enhancing O-GlcNAcylation by siOGA^15^. Moreover, silencing of OGT significantly suppressed the *in vivo* lung metastasis of the 4T1 breast cancer cell line^25^.

The association of increased O-GlcNAcylation and enhancement of tumor growth as well as proliferation of cancer cells have been reported in many cancers^11^,^26^,^27^. O-GlcNAcylation of glucose 6 phosphate dehydrogenase promoted cell proliferation in lung cancer both *in vitro* and *in vivo*^26^. Knockdown of OGT significantly suppressed *in vitro* colony formation and *in vivo* tumor formation of the MDA-MB-231 breast cancer cell line^11^.

Manipulating the level of O-GlcNAcylation using siOGT and siOGA in CCA cells did affect the expression of many metastasis associated genes including MMPs, however, only the expression of MMP7 exhibited a significant response and corresponded well with the level of O-GlcNAcylation in both CCA cell lines. Increasing MMP expression may be the common signaling pathway in response to the elevation of O-GlcNAcylation, as the decrease of MMPs expression after OGT inhibition was repeatedly reported in several cancers such as breast cancer^11^ and prostate cancer^16^. Similarly, the increase of O-GlcNAcylation by OGA inhibition induced the expressions of MMPs in the HepG2 hepatoma cell line^15^.

The mechanism by which O-GlcNAcylation participates in metastasis is not completely understood. Integrated information of O-GlcNAcylated proteins from the dbOGAP database (http://cbsb.lombardi.georgetown.edu/OGAP.html) and the signaling pathways revealed three upstream signaling cascades of MMP activation, PI3K-Akt, Wnt/β-catenin and NF-κB^28^,^29^,^30^,^31^,^32^, that are O-GlcNAc modified. To find out which of these O-GlcNAcylated proteins was involved in the signaling cascade of MMP activation, the activations of Akt, β-catenin and NF-κB, were investigated. The results indicated that suppression of O-GlcNAcylation in siOGT-treated cells significantly reduced the Akt phosphorylation and nuclear translocation of NF-κB. This observation was confirmed by the fact that enhancing O-GlcNAcylation in siOGA treated cells gave the opposite observations with siOGT treated cells. These results suggested Akt and NF-κB to be the upstream O-GlcNAc-modified proteins responsible for MMP activation. Moreover, the essential role of O-GlcNAcylation for nuclear translocation of NF-κB was confirmed by the NF-κB imunocytofluorsecent staining data. These findings are in agreement with previous reports. The importance of O-GlcNAcylation on nuclear translocation of NF-κB and the consequential transcriptional activation has been shown in Caco2 cells^27^,^33^.

The associations of nuclear translocation of NF-κB and the increased migration/invasion and activation of MMP expression observed in siOGA treated cells were further elucidated. DHMEQ, a well-known inhibitor of nuclear translocation of NF-κB, was used as the model. These data clearly showed that DHMEQ had no effect on either cellular O-GlcNAcylation or NF-κB expression but strongly inhibited nuclear translocation of NF-κB and consequently suppressed cell migration/invasion and MMP7 expression. To tie the connection of O-GlcNAcylation and NF-κB, it was next demonstrated that NF-κB was, in fact, O-GlcNAcylated in the study model. Immunoprecipitation experiments using an antibody specific to NF-κB and probed with an antibody against O-GlcNAcylated proteins; and *vice versa* were performed. The results confirmed that NF-κB was O-GlcNAcylated and regulated via manipulation of cellular O-GlcNAcylation.

**Figure 7:**
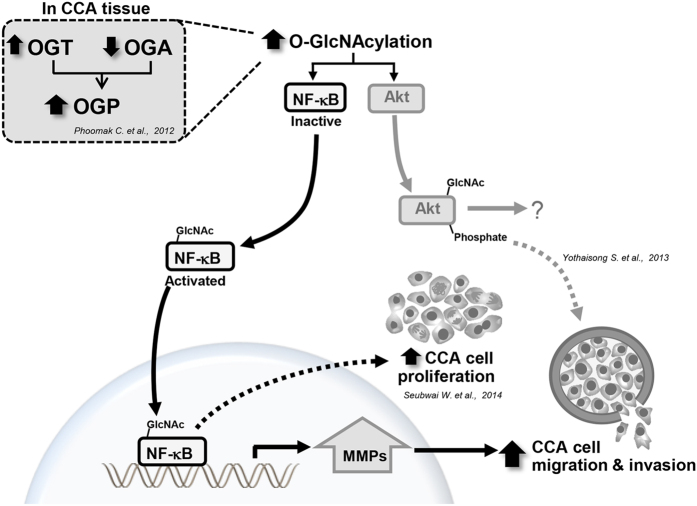
Schematic diagram depicts the mechanism by which O-GlcNAcylation promotes metastasis of CCA cells via modulating the nuclear translocation of NF-κB and MMPs expression. The increase of OGT and decrease of OGA expression leading to the elevation of OGP was found in tumor tissues of CCA patients. The elevation of O-GlcNAcylation activates NF-κB and Akt signaling pathways. As shown in this study, O-GlcNAcylation of NF-κB is necessary for nuclear translocation and the consequential transcriptional activation of the down-stream signaling of NF-κB. Expression of MMP7 was demonstrated to be one of the NF-κB down-stream signaling pathways that is involved with cell migration/invasion. Solid arrows indicate the findings from this study; dashed arrows indicate the findings from other reports.

The results together suggested that elevation of O-GlcNAcylation could enhance migration/invasion ability of CCA cells by sequential reactions as defined in this study: 1) increasing of cellular O-GlcNAcylation increases the O-GlcNAcylation of transcriptional factor NF-κB and the signaling protein (Akt); 2) O-GlcNAc modification activates the nuclear translocation of NF-κB and the phosphorylation of Akt; 3) the transcriptional activation of downstream signaling increases MMP expression and results in 4) the increasing of cell migration and invasion. The schematic outline of this proven mechanism is depicted in Fig. 7.

The findings from this study expand the understanding of O-GlcNAcylation and metastasis in CCA as a whole. Elevation of O-GlcNAcylation in tumor tissues was reported in association with poor survival of CCA patients^20^. The underlining mechanism is shown, in part, via the O-GlcNAcylation/activation of NF-κB and Akt. These two signaling pathways were shown to be essential for metastasis of CCA^21^,^34^. Activation of NF-κB should be a good target for CCA treatment as more than 70% of tumor tissues of CCA patients had high expressions of NF-κB^21^. In addition, inhibition of NF-κB activation significantly induced cell apoptosis in CCA cells, as demonstrated *in vitro* and *in vivo*^21^. Moreover, O-GlcNAcylation of NF-κB, at Thr-322 and Thr-352, has been revealed as an important mechanism to control its nuclear translocation. This specific O-GlcNAcylation may be a novel molecular target inhibitor of NF-κB and consequently CCA metastasis.

Besides the nuclear translocation of O-GlcNAcylated NF-κB, the effect of O-GlcNAcylation on Akt-phosphorylation that may regulate MMP expression and metastasis of CCA should be further studied. Similar observations were also reported in other cancer cells. Enhancement of O-GlcNAcylation by PUGNAc or siOGA induced the phosphorylation of Akt at ser-473 and increased the proliferation of thyroid anaplastic cancer cells^35^ whereas the increase of O-GlcNAcylated Akt conferred chemo-resistance in breast cancer-derived MCF-7 cells^36^.

The modification of proteins by O-GlcNAcylation, especially the transcription factors, is an important process for cell homeostasis. As a consequence, alteration of O-GlcNAcylation participates in the pathological progression of many human diseases including cancers. Understanding O-GlcNAcylation in a particular disease may lead to a novel target for the disease control. In this study, progression of CCA cells, namely in migration and invasion, was enhanced with the increase of O-GlcNAcylation. The observation was tightly regulated by O-GlcNAcylation which was proven in part via the NF-κB signaling pathway. O-GlcNAcylation of NF-κB is essential for nuclear translocation of NF-κB which subsequently activates the transcription of MMPs, the key protease enzymes facilitating metastasis of CCA. Determination of O-GlcNAcylated proteins in highly metastatic CCA cell lines is undertaken to uncover the target proteins modulated by O-GlcNAcylation. O-GlcNAcylation and its products may be the new targets for treatment of CCA with metastasis.

## Materials and Methods

### Cell culture and treatment

CCA cell lines (KKU-213 and KKU-214), were obtained from the Japanese Collection of Research Bioresources (JCBR) Cell Bank, Osaka, Japan. The cell lines were cultured in F-12 Nutrient Mixture (Ham’s F-12) (Gibco, NY) containing 10% FBS and 1% antibiotic-antimycotic. Transient enhancement of O-GlcNAcylation was performed using OGA inhibitor [O-(2-Acetamido-2-deoxy-D-glucopyranosylidenamino)-N-phenylcarbamate; PUGNAc]. Cells were incubated with 20 μM PUGNAc (Sigma Aldrich, St. Louis, MO.) for 1 h before subjecting them to further experiments. Inhibition of nuclear translocation of NF-κB was employed by incubating cells with 2.5 μg/ml dehydroxymethylepoxyquinomicin (DHMEQ)^21^.

### Suppression of OGT and OGA expression by specific-siRNAs

Suppression or enhancing O-GlcNAcylation in CCA cell lines was performed using si-RNA specific to OGT or OGA^15^. CCA cell lines (2 × 10^5 ^cells/well) were cultured in a 6-well plate for 24 h then transfected with 100 pmole of siOGT or siOGA using 2 μg/ml of Lipofectamine 2000 (Invitrogen, NY) according to the recommendations from the manufacturer. Excess transfection complex was removed after 6 h and cells continued to be cultured in 10% FBS in Ham’s F-12 for the subsequent experiments. Control experiments were performed using cells treated with siControl (Negative Control siRNA, 1027310, Qiagen).

### Cell migration and invasion

Migration and invasion were analyzed using the Boyden chamber assay with transwell cell culture inserts (8.0 μm pore size, Corning Incorporated, Corning, NY). Inserts pre-coated overnight with 100 μl of 0.4 mg/ml of basement membrane matrix (BD MatrigelTM, BD biosciences) were used for the invasion assay. siRNA-treated or control cells (4 × 10^4 ^cells/well) were allowed to migrate or invade for 9 h for KKU-213 and 24 h for KKU-214. Non-migrated/invaded cells were scraped out, and the migrated and invaded cells were stained with 0.4% sulforhodamine B in 0.1% acetic acid. Cells were counted under the microscope using a 10X objective lens with 5 microscopic fields/samples. Experiments were performed in duplicate; the data presented are the averages from three independent experiments.

### Cell proliferation

Cell proliferation was measured at 24, 48, 72, and 96 h using the MTT proliferation assay (Moleular probes, Eugene, OR) according to the manufacturer’s guidelines. Briefly, 3-(4,5-Dimethylthiazol-2-yl)-2,5-diphenyltetrazolium bromide (MTT reagent) was added into each well to obtain the final concentration of 0.5 mg/ml. After 4 h incubation, 0.04 N HCl in isopropanol was added to dissolve the insoluble formazan complex and the absorbance at 540 nm was measured. Cell numbers (% of control) were calculated as (OD of treatment × 100)/mean OD of control.

### Nuclear fractionation

The nuclear fraction was extracted as previously described^37^. Briefly, cells were washed by cold PBS, and harvested in cell lysis buffer (10 mM HEPES-KOH, pH 7.9, 1.5 mM MgCl_2_, 10 mM KCl, and 0.5 mM DTT). The nuclear pellets were collected, washed twice with ice cold PBS and lysed with nuclear lysis buffer (20 mM HEPES–KOH pH 7.9, 25% glycerol, 420 mM NaCl, 1.5 mM MgCl_2_, 0.2 mM EDTA, 0.5 mM DTT). Protein concentrations were determined and kept at −20 °C until analysis. Phosphatase inhibitor (PhosStop; Roche Diagnostic, Mannheim, Germany), protease inhibitor (Complete Mini, Roche Diagnostic, Mannheim, Germany), and OGA inhibitor (5 μM PUGNAc) were added at every step.

### SDS-PAGE and Western blot analysis

Total protein was extracted from CCA cell lines using 1% NP-40, 150 mM NaCl, 50 mM Tris-HCl pH7.4 containing phosphatase inhibitor, protease inhibitor, and 5 μM PUGNAc. Protein (30 μg) was solubilized in the sample loading medium (SM; 62.5 mM Tris-HCl pH 6.8, 2% SDS, 5% β-ME, 10% Glycerol, and 0.01% Bromphenol blue), separated by SDS-PAGE according to Laemmli^38^, and electro-transferred onto a PVDF membrane using Bolt and Marhoney transferring buffer (40 mM Tris base, 20 mM sodium acetate, 2 mM EDTA, pH 7.4, 20% methanol, and 0.05% SDS)^39^. Immunodetection of particular proteins was performed using specific monoclonal antibodies as follow; 1:200 of anti-OGT and anti-MMP7 (Santa Cruz Biotechnology, Santa Cruz, CA); 1:400 of anti-β-catenin (BD Transduction Laboratories, New Jersey); 1:1000 of anti-NF-κB (p65) (Santa Cruz), anti-β-tubulin (Santa Cruz, CA), anti-O-GlcNAc (RL2, Pierce Biotechnology, IL), anti-Akt and anti-pAkt (Cell Signaling Technology, Danvers, MA). The immunoreactivity was detected with the ECLTM Prime Western Blotting Detection System (AmershamTM, Buckinghamshire, UK) and analyzed using an ImageQuant LAS 4000 mini image analyzer and ImageQuant™ TL analysis software (GE healthcare, Buckinghamshire, UK).

### RNA extraction and real-time reverse transcriptase polymerase chain reaction

Total RNA was extracted using Trizol reagent (Ambion) and converted to cDNA using the high capacity cDNA Reverse Transcription Kit (Applied Biosystems). Reactions were performed using cDNA converted from 40 ng of total RNA, 0.4 μM of forward- and reverse-primers (Supplementary Table S1), and LightCycle 480® SYBR green I master mix (Roche Diagnostic). PCR was performed in the LightCycle 480® real-time PCR system (Roche Diagnostic, Mannheim, Germany) with a slight modification from that previously described^40^. The annealing at 60 °C for 10 sec and the PCR reaction was ended with a final extension at 72 °C for 10 min. The gene expression level was relatively quantified from duplicated samples using LightCycle 480® Relative Quantification software (Roche Diagnostic). β-2microglobulin (B2M) was used as an internal control^41^.

### Gelatin zymography

The gelatin zymography was performed as previously described^42^. In brief, after 48 h of siRNA treatment, cells in 6 well plates were washed and maintained in 800 μl serum free medium for 24 h then 200 μl of conditioned medium collected and concentrated by a CentriVap centrifugal concentrator (Labconco, Kansas, MO). Samples were reconstituted with 20 μl of 1X sample buffer (62.5 mM Tris-HCl pH 6.8, 2% SDS, 10% glycerol, 0.01% bromphenal blue) and separated by electrophoresis in 10% polyacrylamide gel containing 0.1% gelatin using Leammli buffer. After washing, gelatinase activity was stimulated by incubation overnight in 50 mM Tris PH 7.4, 5 mM CaCl_2_, 1 μM ZnCl_2_, 0.01% NaN_3_ at 37 °C and stained with 0.5% Coomassie blue R250.

### Immunocytofluorescence

After treatment, cells were fixed with 4% paraformadehyde and permeabilized using 0.2% Triton X-100. After blocking the non-specific binding with 5% FBS in PBS, cells were incubated with 1:100 anti-NF-κB (Santacruz) at 4 °C, overnight. The reactivity was detected by incubation with 1:100 anti-rabbit-IgG-FITC (Santa Cruz) for 1 h at room temperature. Nuclei were stained with 1:10,000 Hoechst 33342 (Molecular probe, invitrogen, UK) and the fluorescence signal was observed under the Nikon fluorescence microscope (Nikon Corporation, Tokyo, Japan).

### Immunoprecipitation

Cells were lysed by lysis buffer containing 25 mM HEPES, 10 mM Na_4_P_2_O_7_.10H_2_O, 100 mM NaF, 5 mM EDTA, 2 mM Na_3_VO_4_, 1% Triton X-100, and 5 μM PUGNAc. Cell lysates (385 μg) were immunoprecipitated with 2 μg anti-O-GlcNAc (RL2) at 4 °C, overnight. Protein-G sepharose beads were added and continually rotated for 2 h. After complete rotation, the mixtures were centrifuged at 5,000 rpm for 5 min; the precipitated-beads were subsequently washed 5 times with lysis buffer. After washing, the bound proteins were solubilized with SM-buffer, then subjected to SDS-PAGE and western blotting.

### Statistical analysis

Statistical analysis was performed using GraphPad Prism® 5.0 software (GraphPad software, Inc., La Jolla, CA). Student’s t-test was used to compare the ability of cells on particular functions between treatments and controls. P < 0.05 was considered statistically significant.

## Additional Information

**How to cite this article**: Phoomak, C. *et al*. Mechanistic insights of O-GlcNAcylation that promote progression of cholangiocarcinoma cells via nuclear translocation of NF-κB. *Sci. Rep.* **6**, 27853; doi: 10.1038/srep27853 (2016).

## Supplementary Material

Supplementary Information
