# Supplementary material for: Mechanistic insights of O-GlcNAcylation that promote progression of cholangiocarcinoma cells via nuclear translocation of NF-κB
Source: Sci Rep. 2016 Jun 13;6:27853. doi: 10.1038/srep27853 (PMC4904198; doi:10.1038/srep27853)
Supplement: Supplementary Information [file srep27853-s1.pdf]

Mechanistic insights of O-GlcNAcylation that promote progression of cholangiocarcinoma cells via nuclear translocation of NF-κB

Chatchai Phoomak[1,3], Kulthida Vaeteewoottacharn[1,3], Kanlayanee Sawanyawisuth[1,3], Wunchana Seubwai[2,3], Chaisiri Wongkham[1,3], Atit Silsirivanit[1,3]*, Sopit Wongkham[1,3]*

[1]Department of Biochemistry, Faculty of Medicine, Khon Kaen University, Khon Kaen, 40002, Thailand.

[2]Department of Forensic Medicine, Faculty of Medicine, Khon Kaen University, Khon Kaen, 40002, Thailand.

[3]Liver Fluke and Cholangiocarcinoma Research Center, Faculty of Medicine, Khon Kaen University, Khon Kaen, 40002, Thailand.

Corresponding authors:

Atit Silsirivanit, Ph.D.
Department of Biochemistry,
Faculty of Medicine, Khon Kaen University,
Khon Kaen, 40002, Thailand
Tel/Fax: +66-43-348-386
Email address: atitsil@kku.ac.th

Sopit Wongkham, Ph.D.
Department of Biochemistry,
Faculty of Medicine, Khon Kaen
University, Khon Kaen, 40002, Thailand
Tel/Fax: +66-43-348-386
Email address: sopit@kku.ac.th

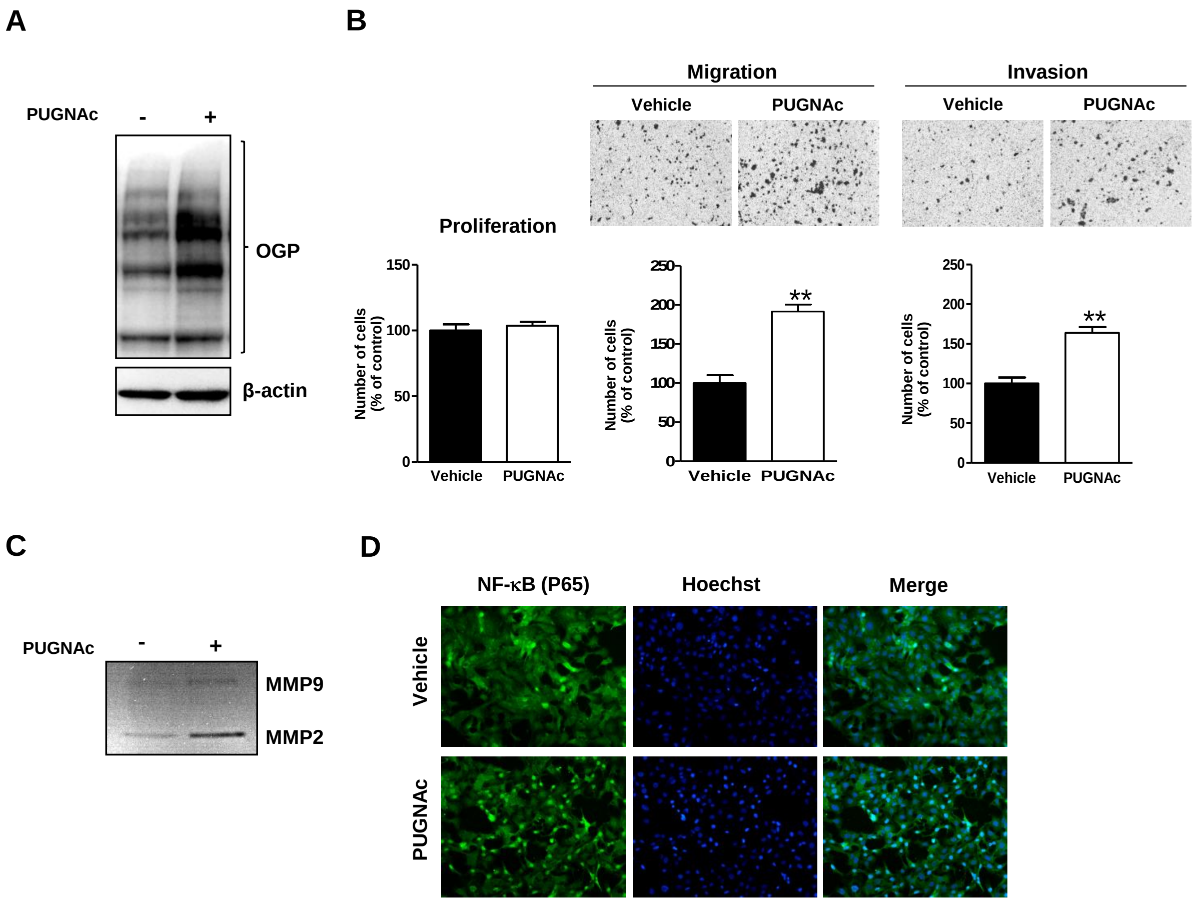

Supplementary Figure S1. Increasing O-GlcNAcylation in KKU-100 using PUGNAc yielded similar effects as siOGA treated cells. The CCA cell line, KKU-100, was treated with PUGNAc, a well-known OGA inhibitor, for 24 h. The effects of O-GlcNAcylation of PUGNAc treated and the control cells were compared on (A) Western blotting of O-GlcNAcylated products (OGP), (B) cell growth, migration and invasion observed at 28 h, (C) MMP2 and MMP9 zymogram and (D) nuclear translocation of NF-κB.

Supplementary Table S1: Primers for determination of genes using real-time PCR

Gene	Ref. No.	Forward	Reverse
CDH1	NM_004360	5'-CTTAGAGGTCAGCGTGTG-3'	5'-AGCAAGAGCAGCAGAATC-3'
CDH2	NM_007664.4	5'-CCTGAGGGATCAAAGCCTGG-3'	5'-ACATGTTGGGTGAAGGGGTG-3'
FN1	NM_002026.2	5'-TGGTGTCACAGAGGCTACTAT-3'	5'-GAAGGGTTACCAGTTGGGGA-3'
ITGA5	NM_002205.2	5'-GATGCCCTACCGAATCCTGC-3'	5'-TGATGATCCACAGTGGGACG-3'
ITGB1	NM_002211.3	5'-GAGTCGCGGAACAGCAGG-3'	5'-AGCAAACACACAGCAAACTGA-3'
MMP2	NM_001127891.1	5'-TGTTAGGCAAGTGACTTCTCAGT-3'	5'-AACAGGTTGCAGCTCTCCTT-3'
MMP3	NM_002422.3	5'-AGACTTTCCAGGGATTGACTC-3'	5'-ACAATTAAGCCAGCTGTTACTCT-3'
MMP7	NM_002423.3	5'-GATAAGCACTGTTCCTCCAC-3'	5'-CACAGTCACACCATAAAGGA-3'
MMP9	NM_004994.2	5'-ACGCACGACGTCTTCCAGTA-3'	5'-CTTGGTCCACCTGGTTCAACT-3'
PAI1	NM_000602.4	5'-CAGGACGAACCGCCAATCG-3'	5'-CAGGTGGGCCACGTAGGATG-3'
SNAI2	NM_003068.4	5'-ACAGCGAACTGGACACACAT-3'	5'-GCGGTAGTCCACACAGTGAT-3'
OPN	NM_000582.2	5'-GCGAGGAGTTGAATGGTG-3'	5'-ATAATCTGGACTGCTTGTGG-3'
TIMP1	NM_003254.2	5'-GCTTCTGGCATCCTGTTGTTG-3'	5'-GGTCTGGTTGACTTCTGGTGT-3'
TIMP2	NM_003255.4	5'-ACCCTCTGTGACTTCATCGTG-3'	5'-CGTCCGGGGAGGAGATGTAG-3'
TWIST1	NM_000474.3	5'-GGAGTCCGCAGTCTTACGAG-3'	5'-TCTGGAGGACCTGGTAGAGG-3'
VCAN	NM_004385	5'-GGCACCTGTTATCCTACTG-3'	5'-GTCTCGGTATCTTGCTCAC-3'
VIM	NM_003380.3	5'-AACTTTTCCTCCCTGAACCTGAG-3'	5'-TCAAGGTCATCGTGATGCTGAG-3'
B2M	AK022379	5'-AAGATGAGTATGCCTGCCG-3'	5'-CGGCATCTTCAAACCTCC-3'

Supplementary Table S2: Relative expression of metastasis associated genes in KKU-213 treated with siOGT or siOGA

**Genes**	**siOGT**	***P***	**siOGA**	***P***
E- cadherin (CDH1)	0.59	0.007	0.72	<0.001
Fibronectin (FN1)	0.97	0.530	1.14	0.315
Integrin-a5 (ITGA5)	0.57	<0.001	1.18	0.014
Integrin-β1 (ITGB1)	0.65	<0.001	1.21	0.006
MMP2	0.69	0.005	1.60	<0.001
MMP3	0.81	<0.001	0.89	0.023
MMP7	0.58	<0.001	1.43	0.015
MMP9	1.50	<0.001	2.81	<0.001
N-cadherin (CDH2)	0.88	0.003	1.07	0.182
Osteopontin (OPN)	1.56	0.002	1.32	0.049
Serpine 1 (PAI-1)	0.72	<0.001	1.67	0.008
Slug (SNAI2)	0.88	0.003	1.15	0.072
TIMP1	0.84	<0.001	0.89	0.249
TIMP2	0.82	<0.001	1.10	0.022
Twist	0.94	0.198	1.06	0.680
Versican (VCAN)	0.95	0.291	0.90	0.049
Vimentin (VIM)	1.11	<0.001	1.28	0.006
